# Diagnostic value comparison of CellDetect, fluorescent in situ hybridization (FISH), and cytology in urothelial carcinoma

**DOI:** 10.1186/s12935-021-02169-3

**Published:** 2021-09-06

**Authors:** Donghao Shang, Yuting Liu, Xiuhong Xu, Zhenghao Chen, Daye Wang

**Affiliations:** 1 Department of Urology, Friendship Hospital, Capital Medical University, Beijing, 100050 China; 2 Department of Pathology, Capital Medical University, Beijing, 100069 China

**Keywords:** Urothelial carcinoma (UC), CellDetect, Fluorescent in situ hybridization (FISH), Cytology

## Abstract

**Background:**

To evaluate the clinical effectiveness of a novel CellDetect staining technique, compared with fluorescent in situ hybridization (FISH), and urine cytology, in the diagnosis of urothelial carcinoma (UC).

**Methods:**

A total of 264 patients with suspicious UC were enrolled in this study. All tissue specimens were collected by biopsy or surgery. Urine specimen was obtained for examinations prior to the surgical procedure. CellDetect staining was carried out with CellDetect kit, and FISH was performed with UroVysion detection kit, according to the manufacturer’s instructions. For urine cytology, all specimens were centrifuged using the cytospin method, and the slides were stained by standard Papanicolaou stain.

**Results:**

In this study, there were 128 cases of UC and 136 cases of non-UC, with no significant difference in gender and age between the two groups. Results for sensitivity of CellDetect, FISH, and urine cytology were 82.8%, 83.6%, and 39.8%, respectively. The specificity of the three techniques were 88.2%, 90.4%, and 86.0%, respectively. The sensitivity of CellDetect and FISH are significantly superior compared to the conventional urine cytology; however, there was no significant difference in specificity among three staining techniques. In addition, the sensitivity of CellDetect in lower urinary tract UC, upper urinary tract UC, non-muscle-invasive bladder cancer (NMIBC), and muscle-invasive bladder cancer (MIBC) were 83.3%, 81.8%, 83.5%, and 72.0%, respectively. The screening ability of CellDetect has no correlation with tumor location and the tumor stage. The sensitivity of CellDetect in low-grade UC and high-grade UC were 51.6 and 92.8%. Thus, screening ability of CellDetect in high-grade UC is significantly superior compared to that in low-grade UC.

**Conclusions:**

CellDetect and FISH show equal value in diagnosing UC, both are superior to conventional urine cytology. Compared to FISH, CellDetect is cost effective, easy to operate, with extensive clinical application value to monitor recurrence of UC, and to screen indetectable UC.

## Background

Urothelial carcinoma (UC) is the second most common urologic malignancy after prostate cancer, which accounts for approximately 90% of all bladder cancer [1]. Upper urinary tract UC is rare in clinical practice and accounts for about 5% of all UC [2]. Although transurethral resection (TUR) is the primary regimen for patients with non-muscle-invasive bladder cancer (NMIBC), the reported five-year overall survival rate has been 90%. Once disease progressed, the 5-year overall survival rate drops to 25–60% for muscle-invasive bladder cancer (MIBC) [3, 4]. With up to 80% recurrence rate, a routine endoscopy surveillance is essential for early tumor detection; UC is considered one of the costliest malignancies in terms of lifetime follow-up [5, 6]. Cystoscopy and ureteroscopy cannot be tolerated by some patients, due to pain and anesthesia. Thus, early noninvasive screening and diagnosis are used for the prognosis of UC patients, imaging and urine cytologic examinations are widely used for detection.

Urine cytology is the most common noninvasive examination for UC detection, however, a large scale study provides further evidence that cytology has low sensitivity for UC detection [7]. In recent years, several urine biomarkers have been discovered for patients with UC, such as fibrinogen degradation product, bladder tumor antigen (BTA) and nuclear matrix protein. Despite the high sensitivity compared with cytology examination, high false-positive rate and poor specificity restricted their clinical application [8]. FISH is also frequently performed in the surveillance of patients with a history of UC [9, 10]. FISH is a multicolor and multitarget examination that has been established for the detection of UC in the urine [11]. Many studies have compared the performance characteristics (sensitivity and specificity) of FISH to conventional cytology, and the results indicated that FISH demonstrated higher sensitivity and similar specificity compared with the conventional urine cytology [12, 13].

CellDetect is a novel cell staining technique by Zetiq Technologies Ltd (Israel) for cancer screening and diagnosis [14]. CellDetect staining system is composed of generic dyes and a unique plant extract (Ficus elastica), the active component of the plant extract was a class of polyphenols present in a variety of plants. It displays a dual color with more cyto-morphological details compared to hematoxylin and eosin staining. In CellDetect stained sections, normal cells generally present blue/green after staining, contrasting with tumor cells present red/purple after staining. Due to its excellent staining characteristics, CellDetect could distinguish normal cells from tumor cells even in a small tumor foci. It has the potential to become one of the most effective examinations for cervical cancer screening and early diagnosis [15].

In this study, we compared the clinical effectiveness of CellDetect, FISH, and urine cytology in the diagnosis of UC. The results showed that CellDetect provided a unique and useful tinctorial clue for the detection of UC. CellDetect used in urine exfoliated cell screen provides an effective technique for early diagnosis of human UC.

## Patients and methods

### Sample collection

A total of 264 patients with suspicious UC were enrolled in the Department of Urology of Beijing Friendship Hospital from January 2020 to March 2021. All subjects had hematuresis, irritative bladder symptom, abdominal pain, and hydronephrosis on the affected side. No patient received neoadjuvant chemotherapy. All tissue specimens were diagnosed through biopsy or surgery. Histological cell type of the resected UC samples was determined by two experienced pathologists, tumor stage was evaluated according to the Union for International Cancer Control (UICC) 2017 TNM classification system, and histological grade was also assessed according to the World Health Organization (WHO) 2004 grading system for UC. Prior to the surgical procedure, urine was obtained for CellDetect, FISH, and urine cytology. On the onset of the study, study protocol approved and provided by the Research Ethics Committee of Beijing Friendship Hospital and informed consent were provided by all patients.

### CellDetect staining

Cell staining was carried out with CellDetect kit (Zetiq Techoogies Ltd., Tel Aviv, Israel), according to the manufacturer’s instruction. Urine sample (minimum of 50 ml) was collected from each patient and smeared onto the slide. After mixing with 10% of trichloroacetic acid, nuclease was stained first with hematoxylin, followed by a differentiation with hydrogen chloride/ethanol. Followed by staining with the red and the green dye in the kit. In between staining, conditioning was performed using the plant extract, and further differentiation was carried out as mentioned above. Normal, inflammatory, and malignant cells can easily be differentiated. Malignant cells show a red nucleus and pink cytoplasm; normal urothelial or squamous epithelial cells show a dark purple or green nucleus and green cytoplasm; inflammatory cells show a purple nucleus and red cytoplasm, and can be distinguished morphologically. Each slide was accessed under a microscope by two cytologists (a third cytologist was on standby for any discrepancies), and assigned two categories according to the best practice of pathology department: negative, and positive [16].

### FISH and cytology

A total of 200 ml urine was collected for FISH and cytology examination. After centrifugation for five minutes at 2000 rpm, two slides were prepared with the ThinPrep^®^5000 processor (Hologic, Inc, Marlborough, MA, USA), one slide was used for FISH, and the other was used for urine cytology stained using Papanicolaou staining. FISH was performed with UroVysion detection kit (Abbott Molecular, Chicago, IL, USA) according to the manufacturer’s instructions. Positivity of the FISH test was determined by the criteria as listed in the package after all cells were evaluated. Abnormal cells of ≥ 4 had chromosomal gain of at least two of the chromosomes 3, 7, or 17; with ≥ 10 tetraploid cells with normal morphology; or ≥ 12 cells with homozygous loss of 9p21 locus were considered positive. All cytology specimens were centrifuged and obtained by cytospin method, and slides were stained by standard Papanicolaou method. For diagnosis of negative or positive UC, refer to CellDetect staining.

### Statistical analysis

Data were collected and compiled using SPSS 16.0. All results were expressed as mean ± standard deviation. Statistical significance was determined by Student’s t-test and Chi-squared test for comparisons among CellDetect, FISH, and urine cytology examination. A *p* value of ≤ 0.05 was considered significant.

## Results

A total of 264 patients, 190 male (72%) and 74 female (28%) with suspicious UC; received CellDetect, FISH, and urine cytology examination. All participants received cystoscopy or ureteroscopy, and pathological results were derived from biopsy, TUR, radical cystectomy, and nephroureterectomy. Baseline characteristics of the study population are listed in Table 1. In this study, there were 128 cases of UC and 136 cases of non-UC, and no significant difference was found in gender and age between the two groups. In patients with UC group, 84 patients with tumor detected in lower urinary tract and 44 patients with tumor detected in upper urinary tract. The tumor grade was low-grade in 31 patients (24.2%), and high-grade in 97 patients (75.8%). In addition, tumor stage enrollment in the study was NMIBC in 103 patients (80.5%), and MIBC in 25 patients (19.5%).

**Table 1:** Baseline characteristics of the study population

	UC	Non-UC	*p*
No.	128	136	
Gender			
Male	90	100	
Female	38	36	0.657
Age			
Range	32–89	35–84	
Average ± SD	67.5 ± 10.2	68.9 ± 13.0	0.741
Tumor location			
Lower urinary UC	84		
Upper urinary UC	44		
Grade			
Low grade	31		
High grade	97		
Stage			
NMIBC	103		
MIBC	25		

**Fig. 1:**
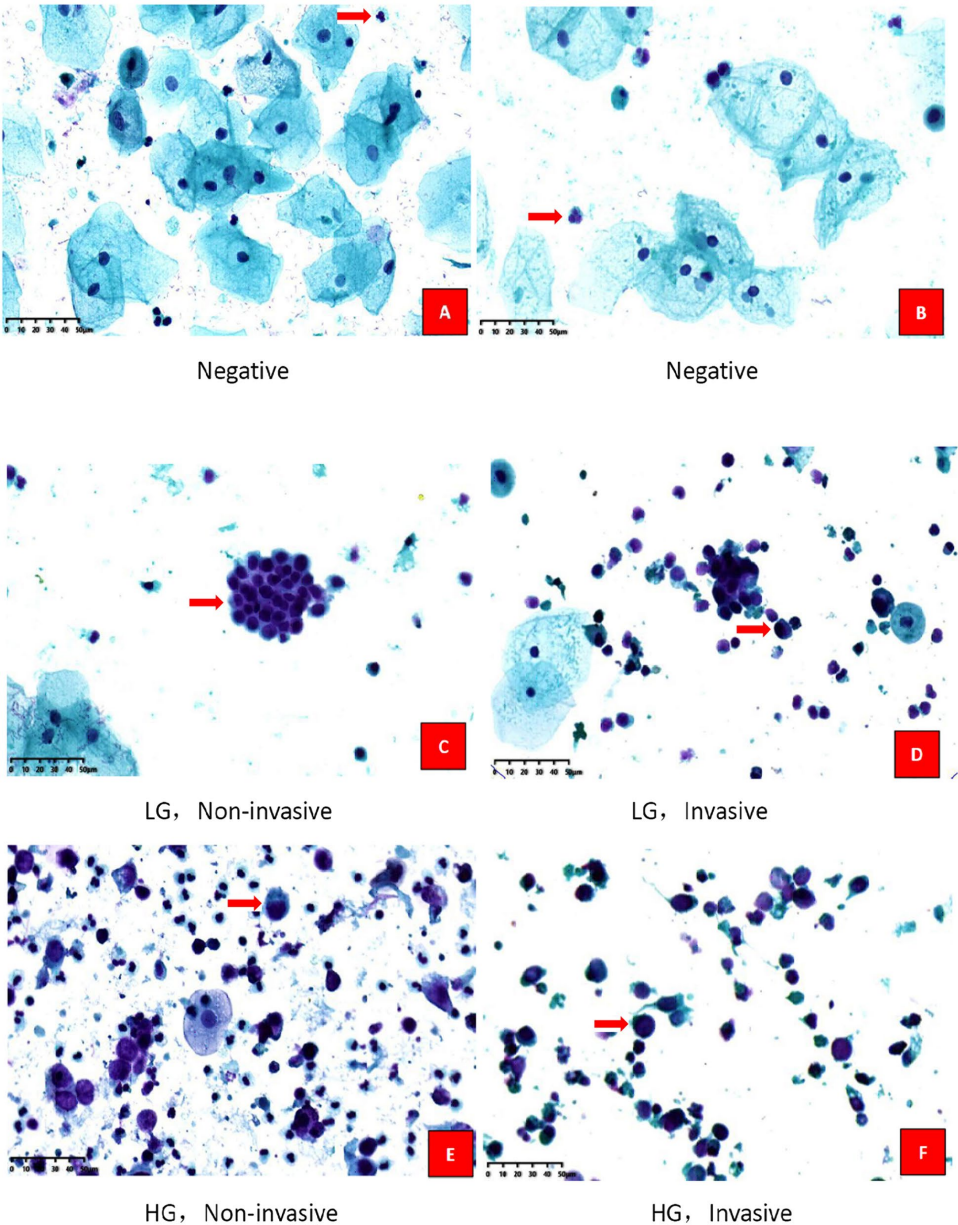
Urine smears stained by CellDetect. **A**, **B** show microscopic features of negative case from non-UC patients. Nuclei of epithelial cells are stained in either green, blue or dark purple, and usually do not show hyperchromasia (with the exception of the pycnotic nuclei of superficial cells). The nuclear/cytoplasmic ratio is low as normal. The inflammatory cells are stained in purple (as arrow direct). And **C**–**F** show UC cells exhibiting reddish-purple nuclei and the nuclear/cytoplasmic ratio is apparently high (as arrow direct). Cytoplasm is either transparent, pink or green. Magnification: ×40, *HG *high-grade, *LG *low-grade

Compared to the conventional urine cytology, CellDetect showed superior features: (1) nuclear/cytoplasmic ratio is maintained; (2) nuclear irregularity is clearly seen; (3) hyperchromic nucleus is evident. The unique plant extract adds color and valuable feature that can easily and accurately detect the suspicious areas. The urine smears stained by CellDetect were shown in Fig. 1.

We evaluated the diagnostic value of CellDetect, FISH and cytology in UC (Table 2). The total sensitivity of CellDetect, FISH, and urine cytology in UC was 106/128 (82.8%), 107/128 (83.6%), and 51/128 (39.8%), respectively. It indicated that CellDetect and FISH have similar sensitivity in the diagnosis of UC, and both examinations are superior to urine cytology. On the other hand, diagnostic specificity of CellDetect, FISH, and urine cytology in UC was 120/136 (88.2%), 123/136 (90.4%), and 117/136 (86.0%), respectively. It indicated no significant difference in the diagnosis of UC. Thus, in the diagnosis of UC, CellDetect has the same screening ability as FISH, and significantly efficient than conventional urine cytology.

**Table 2:** Diagnostic value of CellDetect, FISH and cytology in UC

	CellDetect		FISH		Cytology	
	+	−	+	−	+	−
UC	106	22	107	21	51	77
Non-UC	16	120	13	123	19	117
Sensitivity (%)	82.8		83.6		39.8	
Specificity (%)	88.2		90.4		86.0	

We further evaluated the diagnostic value of CellDetect in UC (Table 3). The total sensitivity of CellDetect in UC of the lower urinary tract and UC of the upper urinary tract were 70/84 (83.3%), and 36/44 (81.8%), respectively (*p* = 0.975). Tumor staging detection on NMIBC, and MIBC by CellDetect were 86/103 (83.5%), and 18/25 (72.0%), respectively (*p* = 0.301). It demonstrated that the screening ability of CellDetect has no correlation with the tumor location and clinical stage. However, total sensitivity of CellDetect in low-grade UC and high-grade UC were 16/31 (51.6%), and 90/97 (92.8%), respectively (*p* < 0.001). Thus, we can conclude that the screening ability of CellDetect in high-grade UC is significantly superior than in low-grade UC.

**Table 3:** Diagnostic value of CellDetect in UC

		CellDetect			
	***n***	+	−	Sensitivity (%)	*p*
Tumor location					
Lower urinary UC	84	70	14	83.3	
Upper urinary UC	44	36	8	81.8	0.975
Grade					
Low grade	31	16	15	51.6	
High grade	97	90	7	92.8	< 0.001
Stage					
NMIBC	103	86	17	83.5	
MIBC	25	18	7	72.0	0.301

## Discussion

Currently, an estimate of 429,000 new cases of UC were diagnosed, with 165,000 deaths per year in the world [17]. China alone has 80,000 new cases of UC with 33,000 deaths per year [18]. Despite the improvement in diagnostic techniques and the progress in surgical therapies, UC has a high recurrence rate and poor prognosis of patients with high-grade UC or MIBC [19]. UC is considered a life-threatening disease, and a routine cystoscopic check is usually performed to screen the recurrence of UC following TUR [20].

Although some noninvasive examinations have been applied to UC detection and screening, the recurrence of UC such as urine cytology and biomarkers, still show low sensitivity to UC diagnosis [21, 22]. Therefore, novel examination and diagnosis innovations are needed for patients with UC. At present, various studies have suggested that FISH is superior to the conventional urine cytology and can improve the diagnosis of UC [23, 24]. FISH can assist in improving UC detection compared to urine cytology. However, FISH also has many disadvantages, which limit its application in UC diagnosis: it requires special supporting equipment; the experimental procedures are relatively complicated and costly; the final decision of UC requires experienced pathologists with UC diagnosis.

CellDetect staining is a unique platform for cancer diagnosis,the proprietary plant extract and dyes enable the color distinction between benign and malignant cells based on staining and morphology. CellDetect was able to spot carcinoma in situ (CIS) cases, even missed by cystoscopy. And for some cases of high-grade UC, the nucleus of the tumor cells shrinked while inflammatory cells enlarged, usually confuses pathologist. However, we found that the nuclei of high-grade UC tend to be smaller, lose their round shape and smooth nuclear membrane, which can be easily observed with CellDetect. A previous study indicated that 94% sensitivity and 89% specificity to detect UC using CellDetect, which had overall superior sensitivity compared to urine cytology [25]. Another study suggested that the sensitivity of CellDetect was 84%, which is more efficient than that of BTA stat in detecting UC [16].

In this study, we compared diagnostic value among CellDetect, FISH, and urine cytology in UC. Our results indicated that CellDetect and FISH have equal-level sensitivity in the diagnosis of UC, and both are significantly superior to conventional urine cytology. However, there was no significant difference in specificity between the three staining techniques. In addition, the sensitivity of CellDetect has no correlation with tumor location and clinical stage. However, the sensitivity of CellDetect in low-grade UC and high-grade UC were 51.6 and 92.8%, respectively, which suggests that the screening ability of CellDetect in high-grade UC is significantly superior to that in low-grade UC. In a previous study on the diagnostic value of FISH and cytology in UC, the sensitivity of FISH in low-grade UC and high-grade UC was 25 and 73%, respectively. The sensitivity of cytology were 36 and 75% [25], respectively. Studies have found that the expression of E-cadherin was down-regulated in high-grade UC, and invasive and distant metastatic UC. This suggests that the reduced expression of adhesion-related protein weakens cell-cell adhesion, and causes tumor cells to detach from the primary site and develop invasion and metastasis [26, 27]. These also explains why the detection rate of high-grade UC is generally higher than that of the low-grade UC by different staining methods used based on the urine exfoliation cytology. In this study, CellDetect displays more cyto-morphological details compared to cytology using pap staining. Due to its excellent staining characteristics, CellDetect could easily distinguish normal cells from UC cells. In addition, compared with FISH, CellDetect has a higher cost performance, a shorter learning cycle, and a relatively simple experimental procedure.

Due to the high recurrence of bladder cancer after TUR, bladder infusion chemotherapy combined with regular cystoscopy is the conventional strategy in the management of lower urinary tract UC. However, cystoscopy has some recognized limitations, such as small or occult tumor lesions that are not easy to visualize and diagnose [28]. The morphological characteristics of CIS under cystoscopy usually appear as erythematous areas, that makes it difficult to distinguish CIS from inflammatory lesions [29]. Some patients cannot tolerate cystoscopy; thus, noninvasive monitoring and diagnostics are extremely important for the screening of patients with lower urinary tract UC. We predicted that the novel urine stain for exfoliated cells will have an expectable clinical application prospect, CellDetect combined imaging examination may play an important role in monitoring recurrence of lower urinary tract UC. Thus, it is worth a further study in the future. Moreover, upper urinary tract UC is defined as a tumor involving the urinary tract between pelvis and ureter. The muscle layers of upper urinary tract are thinner than the bladder, so the UC cells can easily penetrate the muscle layer to form invasive disease, and the prognosis of upper tract UC is poor. Unlike lower urinary tract UC, imaging diagnosis of upper urinary tract UC is usually difficult to determine the diagnosis. Patients generally have poor tolerance to ureteroscopy, which could cause local and distant spread of UC cells. Therefore, early noninvasive screening and diagnosis are extremely important for the prognosis of UC. In this study, the diagnostic value of CellDetect in upper urinary tract UC was equal to that in lower urinary tract UC, which suggests that CellDetect also plays an important role in screening of upper urinary tract UC. The limitation of this study was the sample size was too small, the effect of CellDetect for predicting the recurrence of UC should also be evaluated in future studies.

## Conclusions

In conclusion, CellDetect and FISH show equal value in diagnosing UC, both are superior to that of the conventional urine cytology. CellDetect can obviously improve the ability in monitoring recurrence of UC, and it can be applied in the screening of indetectable UC of the upper urinary tract. CellDetect technique is also cost effective and easier to operate. Thus, it provides an extensive clinical application value in the diagnosis of UC and postoperative follow-up in the future.

## Data Availability

The datasets used and/or analysed during the current study are available from the corresponding author on reasonable request.

## References

[1] Sharma S, Ksheersagar P, Sharma P (2009). Diagnosis and treatment of bladder cancer. Am Fam Physician.

[2] Oosterlinck W, Solsona E, van der Meijden AP, Sylvester R, Bohle A, Rintala E (2004). EAU guidelines on diagnosis and treatment of upper urinary tract transitional cell carcinoma. Eur Urol.

[3] Shariat SF, Karakiewicz PI, Palapattu GS, Lotan Y, Rogers CG, Amiel GE (2006). Outcomes of radical cystectomy for transitional cell carcinoma of the bladder: a contemporary series from the Bladder Cancer Research Consortium. J Urol.

[4] Morgan TM, Clark PE (2010). Bladder cancer. Curr Opin Oncol.

[5] Babjuk M, Bohle A, Burger M, Capoun O, Cohen D, Comperat EM (2017). EAU guidelines on non-muscle-invasive urothelial carcinoma of the bladder: update 2016. Eur Urol.

[6] Svatek RS, Hollenbeck BK, Holmang S, Lee R, Kim SP, Stenzl A (2014). The economics of bladder cancer: costs and considerations of caring for this disease. Eur Urol.

[7] Brimo F, Vollmer RT, Case B, Aprikian A, Kassouf W, Auger M (2009). Accuracy of urine cytology and the significance of an atypical category. Am J Clin Pathol.

[8] Costantini M, Gallo G, Attolini G (2021). Urinary biomarkers in bladder cancer. Methods Mol Biol.

[9] Lotan Y, Shariat SF, Schmitz-Drager BJ, Sanchez-Carbayo M, Jankevicius F, Racioppi M (2010). Considerations on implementing diagnostic markers into clinical decision making in bladder cancer. Urol Oncol.

[10] Maffezzini M, Campodonico F, Capponi G, Canepa G, Casazza S, Bandelloni R (2010). Prognostic significance of fluorescent in situ hybridisation in the follow-up of non-muscle-invasive bladder cancer. Anticancer Res.

[11] Yang T, Li Y, Li J, Liu J, Deng X, Wang G (2018). Diagnostic value comparison of urothelium carcinoma among urine exfoliated cells fluorescent in situ hybridization (FISH) examination, computerized tomography (CT) scan, and urine cytologic examination. Med Sci Monit.

[12] Galvan AB, Salido M, Espinet B, Placer J, Pijuan L, Juanpere N (2011). A multicolor fluorescence in situ hybridization assay: a monitoring tool in the surveillance of patients with a history of non-muscle-invasive urothelial cell carcinoma: a prospective study. Cancer Cytopathol.

[13] Caraway NP, Khanna A, Fernandez RL, Payne L, Bassett RL, Zhang HZ (2010). Fluorescence in situ hybridization for detecting urothelial carcinoma: a clinicopathologic study. Cancer Cytopathol.

[14] Idelevich P, Kristt D, Schechter E, Lew S, Elkeles A, Terkieltaub D (2012). Screening for cervical neoplasia: a community-based trial comparing Pap staining, human papilloma virus testing, and the new bi-functional Celldetect(R) stain. Diagn Cytopathol.

[15] He S, Wang GL, Zhu YY, Wu MH, Ji ZG, Seng J (2014). Application of the CellDetect(R) staining technique in diagnosis of human cervical cancer. Gynecol Oncol.

[16] Davis N, Shtabsky A, Lew S, Rona R, Leibovitch I, Nativ O, et al. A novel urine based assay for bladder cancer diagnosis: multi institutional validation study. Eur Urol Focus. 2018;4(3):388–94. 10.1016/j.euf.2016.10.004 28753799

[17] Ferlay J, Soerjomataram I, Dikshit R, Eser S, Mathers C, Rebelo M (2015). Cancer incidence and mortality worldwide: sources, methods and major patterns in GLOBOCAN 2012. Int J Cancer.

[18] Chen W (2015). Cancer statistics: updated cancer burden in China. Chin J Cancer Res.

[19] Garg M (2015). Urothelial cancer stem cells and epithelial plasticity: current concepts and therapeutic implications in bladder cancer. Cancer Metastasis Rev.

[20] Iwamura H, Hatakeyama S, Sato M, Ohyama C (2018). Asymptomatic recurrence detection and cost-effectiveness in urothelial carcinoma. Med Oncol.

[21] Miyake M, Owari T, Hori S, Nakai Y, Fujimoto K (2018). Emerging biomarkers for the diagnosis and monitoring of urothelial carcinoma. Res Rep Urol.

[22] Kerr PS, Freedland SJ, Williams SB (2020). The current status of molecular biomarkers in patients with metastatic urothelial carcinoma of the bladder. Expert Rev Mol Diagn.

[23] Jin H, Lin T, Hao J, Qiu S, Xu H, Yu R (2018). A comprehensive comparison of fluorescence in situ hybridization and cytology for the detection of upper urinary tract urothelial carcinoma: a systematic review and meta-analysis. Medicine.

[24] Gomella LG, Mann MJ, Cleary RC, Hubosky SG, Bagley DH, Thumar AB (2017). Fluorescence in situ hybridization (FISH) in the diagnosis of bladder and upper tract urothelial carcinoma: the largest single-institution experience to date. Can J Urol.

[25] Lavery HJ, Zaharieva B, McFaddin A, Heerema N, Pohar KS (2017). A prospective comparison of UroVysion FISH and urine cytology in bladder cancer detection. BMC Cancer.

[26] Balci MG, Tayfur M (2018). Loss of E-cadherin expression in recurrent non-invasive urothelial carcinoma of the bladder. Int J Clin Exp Pathol.

[27] Luo Y, Zhu YT, Ma LL, Pang SY, Wei LJ, Lei CY (2016). Characteristics of bladder transitional cell carcinoma with E-cadherin and N-cadherin double-negative expression. Oncol Lett.

[28] Subiela JD, Rodriguez Faba O, Guerrero-Ramos F, Aumatell J, Breda A, Palou J (2020). Carcinoma in situ of the bladder: why is it underdetected?. Curr Opin Urol.

[29] Subiela JD, Rodriguez Faba O, Guerrero Ramos F, Vila Reyes H, Pisano F, Breda A (2020). Carcinoma in situ of the urinary bladder: a systematic review of current knowledge regarding detection, treatment, and outcomes. Eur Urol Focus.

